# Fiber Supplements Derived From Sugarcane Stem, Wheat Dextrin and Psyllium Husk Have Different *In Vitro* Effects on the Human Gut Microbiota

**DOI:** 10.3389/fmicb.2018.01618

**Published:** 2018-07-20

**Authors:** Hasinika K. A. H. Gamage, Sasha G. Tetu, Raymond W. W. Chong, Daniel Bucio-Noble, Carly P. Rosewarne, Liisa Kautto, Malcolm S. Ball, Mark P. Molloy, Nicolle H. Packer, Ian T. Paulsen

**Affiliations:** ^1^Department of Molecular Sciences, Macquarie University, Sydney, NSW, Australia; ^2^Australian Research Council Industrial Transformation Training Centre for Molecular Technologies in the Food Industry, Macquarie University, Sydney, NSW, Australia; ^3^Commonwealth Scientific and Industrial Research Organisation, Sydney, NSW, Australia; ^4^Gratuk Technologies Pty Ltd, Sydney, NSW, Australia

**Keywords:** gut microbiota, dietary fiber supplementation, *in vitro* gut models, 16S rRNA gene, short chain fatty acids, polyphenols

## Abstract

There is growing public interest in the use of fiber supplements as a way of increasing dietary fiber intake and potentially improving the gut microbiota composition and digestive health. However, currently there is limited research into the effects of commercially available fiber supplements on the gut microbiota. Here we used an *in vitro* human digestive and gut microbiota model system to investigate the effect of three commercial fiber products; NutriKane™, Benefiber® and Psyllium husk (Macro) on the adult gut microbiota. The 16S rRNA gene amplicon sequencing results showed dramatic fiber-dependent changes in the gut microbiota structure and composition. Specific bacterial OTUs within the families *Bacteroidaceae, Porphyromonadaceae, Ruminococcaceae, Lachnospiraceae*, and *Bifidobacteriaceae* showed an increase in the relative abundances in the presence of one or more fiber product(s), while *Enterobacteriaceae* and *Pseudomonadaceae* showed a reduction in the relative abundances upon addition of all fiber treatments compared to the no added fiber control. Fiber-specific increases in SCFA concentrations showed correlation with the relative abundance of potential SCFA-producing gut bacteria. The chemical composition, antioxidant potential and polyphenolic content profiles of each fiber product were determined and found to be highly variable. Observed product-specific variations could be linked to differences in the chemical composition of the fiber products. The general nature of the fiber-dependent impact was relatively consistent across the individuals, which may demonstrate the potential of the products to alter the gut microbiota in a similar, and predictable direction, despite variability in the starting composition of the individual gut microbiota.

## Introduction

Trillions of microorganisms reside in the human large intestine, which is collectively referred to as the gut microbiota (Cani et al., 2016). The gut microbial composition is shaped by exogenous and endogenous factors, and interacts with the host metabolism and physiology (Kovatcheva-Datchary et al., 2015). The adult gut microbiota is usually dominated by the phyla *Firmicutes* and *Bacteroidetes* (Bäckhed et al, 2005; Arumugam et al., 2011) while *Actinobacteria, Proteobacteria* and *Verrucomicrobia* constitute minor proportions of the bacterial populations (Eckburg et al., 2005; Lozupone et al., 2012). Compositional and functional alterations of the gut microbiota have been associated with various inflammatory and metabolic diseases such as obesity (Turnbaugh et al., 2009), type 2 diabetes (T2D) (Karlsson et al., 2013), type 1 diabetes (T1D) (Knip and Siljander, 2016), and inflammatory bowel disease (IBD) (Frank et al., 2007).

Diet has been shown to impact the composition and activities of the gut microbiota (Conlon and Bird, 2015; Fontana and Partridge, 2015). The overall structure of the gut microbiota has been reported to respond within a day to short-term consumption of entirely animal or plant based diets (David et al., 2014). There is also evidence that individual dietary preferences correlate to some degree with longer-term gut microbiota composition (Wu et al., 2011). Previous studies have shown that bacteria in the genus *Bacteroides* are more dominant in the gut microbiota of people consuming high levels of protein and animal fat, while *Prevotella* are dominant in the gut microbiota of frequent fiber and carbohydrate consumers (Wu et al., 2011). Similar observations have been made in a number of studies that looked at different communities of people who consume diets rich in fiber in comparison to diets low in fiber (De Filippo et al., 2010; Yatsunenko et al., 2012; Lin et al., 2013; Schnorr et al., 2014; O'Keefe et al., 2015).

Modulation of the gut microbiota using dietary components is potentially therapeutically useful (Doré and Blottiere, 2015; Wu et al., 2015). Prebiotics are generally non-digestible by humans, but are fermented by the gut microbiota to yield energy and metabolic end products of microbial fermentation, such as short chain fatty acids (SCFAs) (Tremaroli and Backhed, 2012; Slavin, 2013; Janssen and Kersten, 2015). SCFAs, mainly acetate, propionate and butyrate have established roles in host physiology. These compounds provide an energy source that accounts for up to 10% of daily caloric value (Sonnenburg and Backhed, 2016). They act as modulators of autophagy in colonocytes; as precursors and regulators of cholesterol, fatty acids and glucose; as well as activators of anti-inflammatory effects, tumor suppression and production of the hormone leptin (Koppel et al, 2016).

The most commonly studied prebiotics are dietary fiber, which include carbohydrates such as cellulose, xylan, resistant starch, pectin, inulin and mannan (Slavin, 2013). Fermentation of dietary fiber by the gut microbiota and concomitant effects on human health has been investigated in the context of conditions such as IBD, T2D and obesity (Rastall and Gibson, 2015). For example, studies have shown an increase in the abundance of bifidobacteria upon consumption of inulin, short chain fructooligosaccharides (FOS) or galactooligosaccharides (GOS) (Yasmin et al., 2015) and the ability of these dietary fibers to reduce inflammatory markers associated with obesity and T2D (Vulevic et al., 2013; Dehghan et al., 2014). *In vivo* studies conducted using inulin and various oligofructoses have shown an inhibition of animal and human pathogenic bacterial groups and increase in bifidobacteria and SCFAs (Saad et al., 2013). Several recent studies have also employed *in vitro* models to compare the effects of introducing pectin, inulin (Johnson et al., 2015; Chung et al., 2016), and wheat dextrin (Hobden et al., 2013) on the gut microbiota. These studies have demonstrated the potential of the fiber additions to enrich specific members of the genus *Bacteroides* and phylum *Firmicutes* (Hobden et al., 2013; Johnson et al., 2015; Chung et al., 2016).

Dietary polyphenols and antioxidants have also been studied for their ability to beneficially change gut microbial composition and functions (Hervert-Hernández and Goñi, 2011; Cheng et al., 2016) and therefore also have prebiotic potential. Polyphenols from various fruits and tea have been shown to inhibit the growth of pathogens and maintain the growth of *Lactobacillus, Bifidobacterium, Lachnospiraceae*, and *Eubacterium rectale* (Hervert-Hernández and Goñi, 2011; Sheflin et al., 2016). Several other studies have indicated the ability of the antioxidant action to be delivered to the gut epithelia resulting in a reduction of inflammation (González-Gallego et al., 2010; González et al., 2011; Bonaccio et al., 2016) and improvement in tissue recovery in IBD patients (Shapiro et al., 2007).

Utilization of *in vitro* models of the human gut microbiota to investigate the impact of dietary interventions on the microorganisms provide powerful information for proof of concept studies prior to *in vivo* validation (Payne et al., 2012; Williams et al., 2015). *In vitro* models facilitate frequent sampling, increase the reproducibility and provide a simplified model to focus on the gut microbiota without issues such as host variability, ethical approval and volunteer compliance (McDonald et al., 2013). Various *in vitro* models of the gut microbiota have been used to examine the effects of prebiotics (Bussolo de Souza et al., 2014; Chung et al., 2016), probiotics (Cordonnier et al., 2015), diet (Condezo-Hoyos et al., 2014), and dietary modulations (Aguirre et al., 2016) on the gut microbiota and its metabolites.

The recommended daily individual intake of dietary fiber in many countries ranges from 25 to 30 g/day (McRorie, 2015), however, increasing amounts of data show that this requirement is poorly met, especially in many western countries (Cordain et al., 2005). Many commercially available fiber supplements are marketed as a means of bridging this gap in dietary fiber intake. However, to date only a few studies have directly examined commercially available dietary fiber products for prebiotic potential (Hobden et al., 2013; Grimaldi et al., 2016).

In this study, we investigated the effect of three commercially available fiber products in the Australian market, namely, NutriKane™, Benefiber® and Psyllium husk (Macro) on the human gut microbiota using an *in vitro* model system. Alterations in microbial community composition, as well as the production of metabolites such as SCFAs were examined. The chemical composition, antioxidant potential and polyphenolic content of the products were also determined.

## Materials and methods

### Compositional analysis of fiber products

Fiber products used in this experiment are derived from dried whole sugarcane and pectin from apple and citrus fruits (NutriKane), wheat dextrin (Benefiber) and psyllium husk (Macro Organic Psyllium husk).

The chemical composition of each fiber product was determined using the following protocols. Total Nitrogen content was measured by the Dumas method with a Series II CHNS/O Analyzer 2400 (Perkin Elmer, Australia). Protein content was calculated by multiplying the nitrogen content by a factor of 6.25 (Ulsemer et al., 2016). Fat content was determined by Soxhlet extraction according to the AOAC Method 945.16. Dietary fiber was determined enzymatically according to the AOAC method 985.29. Insoluble and soluble dietary fiber content was determined according to AOAC 991.43. Acid insoluble lignin was measured gravimetrically following acid hydrolysis (Willför et al., 2009). Monosaccharides were quantified using acetylated samples on a Shimadzu 17A gas chromatograph with flame ionization detection (GC-FID). Quantitation was performed by acetylation of a mixture of monosaccharide standards and 2-deoxy-D-glucose as an internal standard, which was added to all samples at 100 ppm concentration to allow calculation of response factors (full chemical composition and ingredient list provided in Table 1).

**Table 1:** The chemical composition and nutritional profile of fiber products **(A)** The chemical composition of each fiber product determined as described in the Methods section. Values are expressed as g/100 g total weight, unless not detected (ND). The mean ± SD is presented for each compound (n = 3), and **(B)** ingredients and nutritional profile of each product according to the information on the packaging.

**Compound**	**NutriKane**	**Benefiber**	**Psyllium husk**
**(A)**			
Nitrogen	0.08 ± 0.01	ND	0.24 ± 0.01
Protein	0.54 ± 0.04	ND	1.50 ± 0.09
Fat	1.17 ± 0.09	0.02 ± 0.04	0.46 ± 0.15
Total dietary fiber	83.94 ± 0.60	10.31 ± 1.19	77.24 ± 1.18
Insoluble dietary fiber	86.65 ± 1.95	ND	71.74 ± 0.87
Soluble dietary fiber	ND	5.64 ± 1.14	ND
Lignin	20.23 ± 1.08	ND	4.69 ± 0.21
Rhamnose	ND	ND	2.39 ± 0.03
Arabinose	5.83 ± 0.45	1.27 ± 0.2	46.8 ± 0.49
Xylose	31.8 ± 2.47	0.19 ± 0.01	24.1 ± 0.25
Mannose	1.54 ± 0.12	21.3 ± 0.22	4.24 ± 0.04
Glucose	17.3 ± 1.34	74.5 ± 0.78	11.2 ± 0.12
Galactose	0.74 ± 0.06	ND	2.09 ± 0.16
**Fiber supplement**	**NutriKane**	**Benefiber**	**Psyllium husk**
**(B)**			
Ingredients	Sugarcane (sucrose removed)	100% wheat dextrin (derived from wheat)	100% organic psyllium husk
	Pectin (from apple and citrus fruits)		
Dietary fiber content per 100 g	55.2 g	83 g (soluble fiber)	90 g
Nutritional information (average quantity per 100 g)	Energy 784 kJ	Energy 913 kJ	Energy 759 kJ
	Protein 0.8 g	Protein < 1 g	Protein 1.3 g
	Fat total 0.1 g	Fat total < 1 g	Fat total < 1 g
	Saturated 0.1 g	Saturated < 1 g	Saturated < 1 g
	Carbohydrate 6.6 g	Carbohydrate 14.2 g	Carbohydrate < 1 g
	Sugars 4.5 g	Sugars < 1 g	Sugars < 1 g
	Dietary fiber 55.2 g	Dietary fiber (total) 83 g	Dietary fiber 90.1 g
	Sodium 15 mg	Sodium < 5 mg	Sodium 17 mg
	Gluten ND		
	Chromium 391 μg		
	Potassium 5.7 g		

### *in vitro* digestion of fiber supplements

All enzymes and reagents were purchased from Sigma Aldrich, Australia, unless otherwise stated. Gratuk Technologies Pty Ltd, Australia, provided NutriKane. Benefiber and Psyllium husk (Macro) were purchased from a local Australian supermarket.

Each of the three fiber products and a sterile water (Milli-Q, Millipore, Australia) sample as the no added fiber control were processed by a simulated oral, gastric and small intestinal digestion as described previously (Minekus et al., 2014). According to this protocol all enzymatic treatments were performed at 37°C, samples were first incubated with human salivary α-amylase (75 UmL^−1^) for 2 min at pH 7, followed by porcine pepsin (2,000 UmL^−1^) for 2 h at pH 3. The small intestine digestion was performed for another 2 h with the following enzymes: porcine trypsin (100 UmL^−1^), bovine chymotrypsin (25 UmL^−1^), porcine pancreatic lipase (2,000 UmL^−1^), porcine pancreatic colipase (2:1 colipase to lipase molar excess) and bile salts (10 mM) at pH 7. Samples were frozen at −80°C and freeze dried, following digestion.

### Preparation of the basal medium

A basal media was designed to simulate human large intestine conditions (Gamage et al., 2017). The composition of the basal medium per liter was: Peptone 0.5 g, yeast extract 0.5 g, NaHCO_3_ 6 g, Hemin solution (0.05% (w/v) Hemin, and 0.2% (w/v) NaOH) 1 mL, L-cysteine HCl 0.5 g, Bile salts 0.5 g, Tween 80 2 mL, Resazurin solution [0.1% (w/v)] 1 mL, Vitamin stock (Scheifinger et al., 1974) 1 mL, K_2_HPO_4_ 0.228 g, KH_2_PO_4_ 0.228 g, (NH_4_)_2_SO_4_ 0.228 g, NaCl 0.456 g, MgSO_4_ 0.0456 g, CaCl_2_.2H_2_O 0.0608 g, and 1 mL trace mineral solution (Balch et al., 1979) with additional NiSO_4_.6H_2_O (0.1 g/L), Na_2_SeO_4_ (0.19 g/L) and Na_2_WO_2_.2H_2_O (0.1 g/L). The pH of the medium was adjusted to 7.0 ± 0.2.

Preparation of the basal medium and subsequent culturing was conducted under strict anaerobic conditions using a 5% hydrogen and 95% carbon dioxide anaerobic chamber (COY Lab products, Australia). The anaerobic basal medium was aliquoted into airtight glass vials with rubber stoppers and aluminum lids prior to sterilization.

### Collection and preparation of fecal inocula

This study was carried out in accordance with the recommendations of the “National Statement on Ethical Conduct in Human Research 2014” (National Health and Medical Research Council of Australia). The protocol and experimental procedures were reviewed and approved by Macquarie University Human Research Ethics Committee (Medical Sciences, Reference number 5201400595). All participants were provided with a participant information and consent form. To secure anonymity written consent was not obtained, and participation was voluntary.

One fecal sample each was collected from six healthy volunteers (3 male and 3 female) aged 20–60 years, who had not taken antibiotics in at least 3 months, had no history of gastrointestinal diseases and were on a nonspecific omnivorous diet (metadata provided in Supplementary Table S1).

Fresh fecal samples were collected in a sterile container and immediately placed in an anaerobic jar (Anaero jar, Oxoid Limited, UK) with an Anaerogen sachet (Oxoid, UK) and an Oxoid anaerobic indicator (BR0055B, Oxoid, UK). Samples were transported to the laboratory anaerobically and processing occurred within 2 h of collection. Fecal slurries were prepared from individual samples by homogenizing in anaerobic sterile basal medium and filtering through a sterile Nylon mesh cloth (985 μm). This was conducted under strict anaerobic conditions as used for basal media preparation.

### *in vitro* fermentation of fiber supplements

*In vitro* digested and freeze-dried samples of NutriKane, Benefiber and Psyllium husk were added into separate vials with sterile anaerobic basal medium, the final fiber product concentration was maintained at 1% (w/v). A control sample was run in parallel with no added fiber. Each of these vials was then inoculated with filtered fecal homogenate to obtain a final concentration of 2% (w/v) in a final volume of 50 mL (1.0 g feces per vial). Experiments were performed in triplicate for each of the six fecal samples (details of the experiment design are provided in Supplementary Figure S1).

Culture vials were incubated anaerobically at 37°C with agitation (100 rpm). Cultures were left without agitation for 5–10 min, to allow the solids to settle, prior to collecting 2 mL aliquots from the top liquid fraction at 0, 24, and 48 h. Harvested samples were stored at −80°C immediately prior to further analysis. The pH of the cultures at 48 h was measured using pH indicator strips universal pH 0–14 and 4.5–10 (Dosatest, VWR, Australia).

After collecting the liquid fraction samples at 48 h, insoluble fiber biomass (fiber fraction) was separated from each vial with fiber products by centrifugation at 100 × g for 5–15 min. Separated fiber fraction samples were resuspended in Phosphate-buffered saline (PBS) prior to dissociation of tightly adherent microorganisms as previously described (Rosewarne et al., 2011). According to this protocol the insoluble fiber fraction was mixed with a 1:2 (w/v) acid butanol solution [0.1% (v/v) Tween 80, 1% (v/v) methanol and 1% (v/v) tert-butanol, at pH 2.0]. Harvested microbial cells were stored in PBS at −20°C prior to DNA extraction.

### Analysis of the microbial composition

Microbial cells from the liquid fraction samples were harvested by centrifugation at 20,238 × g for 15 min. Harvested cells from the liquid and fiber fraction samples were used for bacterial DNA extraction using a FastDNA spin kit (MP Biomedicals, Australia) according to the manufacturer's instructions. The lysing matrix in the kit was replaced by matrix E (MP Biomedicals, Australia) according to previously published protocols (Gillings, 2014). The V4 region of 16S rRNA gene was amplified using a Five prime hot master mix (5 prime, VWR, Australia) with a final primer concentration at 0.2 μM in a final volume of 25 μL. The PCR was performed with 30 cycles at 94°C for 45 s, 50°C for 60 s, and 72°C for 90 s using 515 forward (5′-GTGCCAGCMGCCGCGGTAA-3′) and 806 reverse (5′-GGACTACHVGGGTWTCTAAT-3′) primers with custom barcodes for Illumina MiSeq sequencing (Caporaso et al., 2011, 2012). Fiber and liquid fraction samples were randomly allocated to libraries. The resulting amplicons were quantified using Quant-iT™ PicoGreen® (Invitrogen, Australia) and equal molar amounts of barcoded amplicons from each sample were pooled, gel purified (Wizard® SV gel and PCR clean up system, Promega, Australia) and sequenced on an Illumina MiSeq platform (2 x 250 bp paired-end sequencing) at the Ramaciotti Centre for Genomics, Australia.

Raw sequence data was processed using Quantitative Insights Into Microbial Ecology (QIIME) software (version 1.9.0) (Caporaso et al., 2010). Reads with full length and high quality (-q 19 and with other default parameters) were used to pick Operational Taxonomic Units (OTUs) at 97% similarity using an open reference protocol against the Greengenes (version 13_8) database (DeSantis et al., 2006).

Liquid fraction samples (*n* = 216, 3 fiber products and a no added fiber control × 6 biological samples × 3 time-points × 3 technical replicates) resulted in a total of 21,052,381 reads (mean 97, 464 ± 25,865) prior to filtering out the OTUs with < 0.005% reads. Reads per sample was rarefied to 38,249 reads (four samples failed to meet this requirement and therefore, were eliminated from further analyses, at least two technical replicates remained for each condition) prior to further statistical analyses.

Fiber fraction samples (*n* = 54, 3 fiber products × 6 biological samples × 3 technical replicates) were analyzed with liquid fraction samples (*n* = 54) at 48 h and this analysis resulted in 9,276,308 reads (mean 85,891 ± 32,494). Reads per sample was rarefied to 12,864 reads prior to statistical analysis, following filtering out OTUs with < 0.005% reads (two samples failed to meet this requirement and therefore were eliminated from further analyses, however at least two technical replicates remained for each condition).

Rarefied and filtered OTUs abundances (Log (x+1) transformed) were used for statistical analyses using the PRIMER-7 software package (Clarke and Gorley, 2015), default parameters on PRIMER-7 were used unless otherwise stated. Permutational Multivariate Analysis of Variance (PERMANOVA) and pairwise tests were conducted using PERMANOVA+ (Clarke et al., 2014) to investigate differences in the microbial community structure in each sample. Type III sums of squares with 9999 permutations were used to determine the *P*-values. Non-metric multi-dimensional scale (nMDS) plots were constructed to visualize the differences in the community structure in each biological sample based on Bray-Curtis similarity of Log (x+1) transformed values of the abundance of the OTUs. The Shannon diversity index, Simpson's evenness index and Chao1 index per liquid fraction sample (*n* = 212) was also determined using PRIMER-7.

Similarity Percentages (SIMPER) analyses with a 5% cut off for low contributions was used to determine the OTUs with significant differences in each treatment using PRIMER-7. Distinct phylotypes (bacterial families and OTUs) between each fiber product and no added fiber control at 48 h were identified using LEfSe analyses (online Galaxy version 1.0) (Segata et al., 2011). LEfSe analysis was conducted with treatment conditions as subject (no subclasses) and with all other default parameters. The significantly differentially abundant OTUs between the fiber adherent and liquid fraction microbiota were also determined using LEfSe analyses. Analyses were conducted with fractions in each product as subject (no subclasses) and with all other default parameters.

### Quantification of SCFAs

The supernatants of the liquid fraction samples (500 μL) collected at 0, 24, and 48 h were spiked with an internal standard (4-methyl valeric acid). This was further diluted in a 70% (v/v) ethanol and 0.1% (v/v) trifluroacetic acid (TFA) solution to obtain a final concentration of the internal standard at 100 ppm. The solution was vortexed then filtered through a 0.2 μm membrane filter (Millipore, Australia). Analysis was performed using a GC-FID (Shimadzu GC-17A). Samples were separated on a 30 m × 0.25 × 0.5 μm i.d. HP-INNOWax fused silica column (Hewlett-Packard, Australia) as per the manufacturer's instructions. GC-FID analysis for each sample was performed in three technical replicates (*n* = 636). The concentrations of SCFAs are reported in mmolL^−1^ per gram of feces.

### Quantification of antioxidant potential and polyphenol content

Total Polyphenolic Content (TPC) was determined as previously described (Singleton and Rossi, 1965). Briefly, 20 μL of sample was mixed with 1.58 mL of water and 100 μL of the Folin-Ciocalteu reagent. After 6 min of incubation, the solution was mixed with 300 μL of 7.5% (w/v) Na_2_CO_3_ and left to stand for 2 h. Gallic acid standards ranged from 25 to 500 mg/L. Absorbance was read at 765 nm and results were reported in mg of Gallic acid per liter.

Ferric reducing antioxidant power (FRAP) was performed as previously described (Benzie and Strain, 1996). The FRAP reagent was prepared by mixing 300 mM acetate buffer (pH 3.6), 10 mM 2,4,6-tripyridyl-s-triazine solution and 20 mM FeCl_3_ in a 10:1:1 ratio. 20 μL of sample was mixed with 0.2 mL of water and 1.8 mL of FRAP reagent and incubated at 37°C for 10 min. Ferrous sulfate standards ranged from 125 to 2,500 μM. The absorbance was read at 593 nm and results reported in millimolar ferric ions converted to the ferrous form per liter.

### Statistical analysis

D'Agostino-Pearson omnibus normality tests were performed and Kruskal-Wallis test with Dunn's multiple comparisons test or Tukey's multiple comparison test was used where appropriate to determine statistically significant differences. Significant differences in the Log (x+1) transformed abundance of the OTUs identified from the SIMPER analysis, Shannon diversity indices, Simpson's evenness indices, pH measurements, concentration of SCFAs, TPC and FRAP measurements were determined between each fiber product and no added fiber control at 24 and 48 h using GraphPad Prism (version 7) software (GraphPad Software, La Jolla California, USA). Biological samples were analyzed independently. The correlations between the relative abundance of the bacterial families, SCFA concentrations, TPC and FRAP measurements were determined using Spearman's correlation analyses (two-tailed test) using GraphPad Prism (version 7) software.

## Results

Samples of three commercially available fiber products; NutriKane, Benefiber and Psyllium husk (Macro), with varying chemical composition (Table 1), were chosen to investigate the impact of fiber supplementation on the human gut microbiota *in vitro*. Each fiber product was subjected to a series of pH-controlled enzyme treatments to simulate human digestion, and the effect of each on the human gut microbiota was examined in an *in vitro* system with an anaerobic basal medium, which simulates conditions in the human large intestine. Fecal material obtained from six healthy adults as independent biological samples were inoculated separately into the basal medium (metadata provided in Supplementary Table S1). For each biological sample, four treatments were applied, this included three fiber products and one “no added fiber” control (details of the experiment design are provided in Supplementary Figure S1).

The anaerobic cultures for all tested fiber products with each fecal inoculum produced visually detectable gas by 24 h, indicating that the microbiota was metabolically active. At 48 h the pH of the culture vials with Benefiber significantly reduced (*P* < 0.001) compared to the samples with NutriKane, Psyllium husk or no added fiber control, which maintained the pH at 7.0 ± 0.5 in the buffered media (Supplementary Figure S2).

Samples were collected at 0, 24, and 48 h from the liquid fraction, and at 48 h the insoluble fiber fraction was additionally sampled. The 16S rRNA gene amplicons were sequenced from each sample. A total of 21,052,381 reads were generated for the liquid fraction samples, and after filtering and rarefaction a total of 8,261,784 reads were used for further analyses. A total of 4,400,597 reads were generated for the fiber fraction samples, and after filtering and rarefaction a total of 681,792 reads were used for further analyses.

### Effects of fiber addition on microbial community structure and diversity

**Figure 1:**
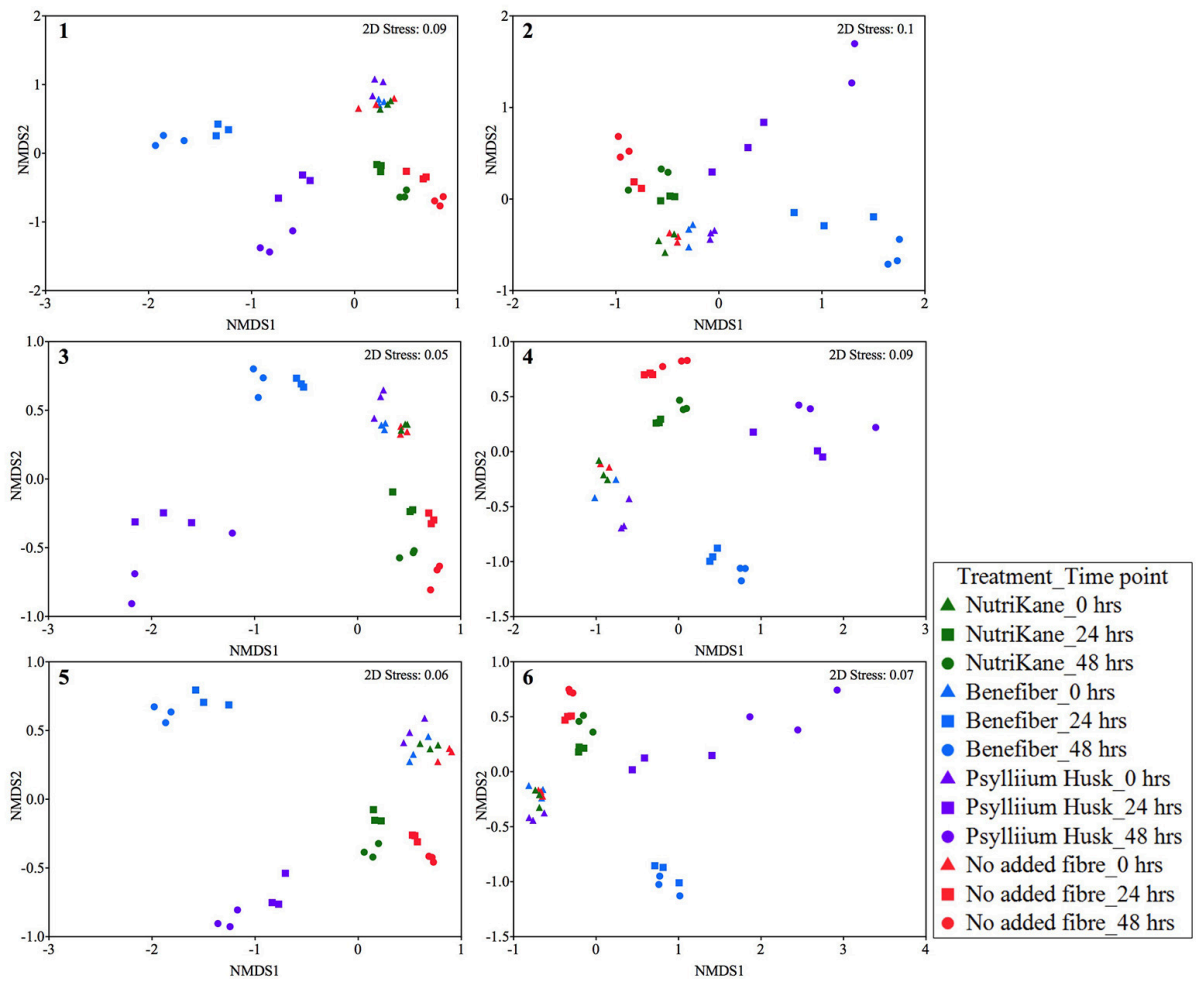
Ordination of the gut microbiota in each biological sample (sample 1-6) at 0, 24, and 48 h. Data is shown as Bray-Curtis similarity of Log (X+1) relative abundance based nMDS plots. At 0 h (triangles) all samples group together. Fiber-dependent shifts were observed at 24 (squares) and 48 (circles) hours in all the treatments. NutriKane (green), Benefiber (blue) and Psyllium husk (purple) had different communities to the no added fiber control (red) while samples with Benefiber and Psyllium husk showed the most dramatic shifts compared to the no added fiber control and other treatments.

To determine the impact of different fiber products on the microbiota in the liquid fraction, statistical analyses were performed to compare the bacterial community structure of samples over time and between treatment conditions. We observed fiber-dependent changes in the bacterial community structure over time based on Bray-Curtis similarity nMDS plots for each biological sample (Figure 1) and PERMANOVA tests. Fiber product-mediated shifts in the gut microbiota structure showed very similar trends upon each treatment at 24 and 48 h across biological samples (Figure 1 and Supplementary Figure S3). For all individuals, supplementation with NutriKane resulted in a significantly different community structure at 24 h compared to at 0 h (*P* < 0.05). At 48 h this shift was more pronounced (*P* < 0.005, comparing 0 and 48 h). Clear shifts in the microbial community structure were observed in the nMDS plots upon addition of NutriKane compared to the no added fiber samples at both 24 and 48 h (Figure 1), however, these differences were not statistically significant based on PERMANOVA tests. Addition of Benefiber and Psyllium husk resulted in very dramatic changes in the community structures. Both the products resulted in significant differences (*P* < 0.001) in the community structure at 24 and 48 h compared to that of the no added fiber control and community at 0 h (*P* < 0.001).

Ordination of the gut microbiota of all samples (*n* = 212) showed significant fiber addition-mediated changes in the community structure common across the biological samples (Supplementary Figure S3). At 0 h all samples grouped according to the individual fecal inoculum, rather than the treatment condition, indicative of the individual variation in the gut microbial composition of the volunteers.

**Figure 2:**
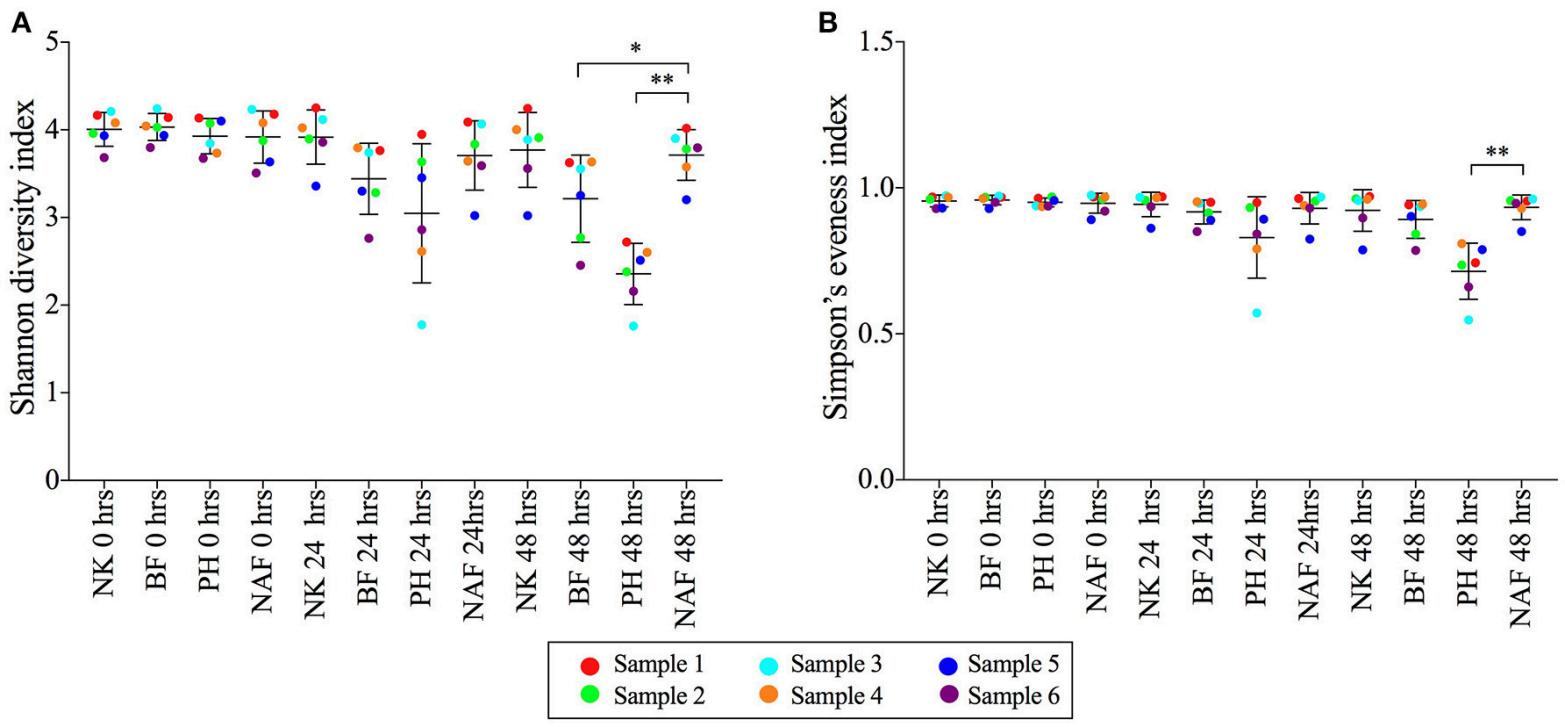
The **(A)** Shannon diversity indices and **(B)** Simpson's evenness indices of microbial communities from each treatment at 0, 24, and 48 h. Data is shown as mean ± SD for samples with NutriKane (NK), Benefiber (BF), Psyllium husk (PH) and no added fiber control (NAF) at 0, 24, and 48 h. Biological samples (sample 1–6) are indicted by color-coded dots as shown in the key. Significance was determined using Kruskal-Wallis test with Dunn's multiple comparisons (*P < 0.05, **P < 0.01).

The microbial diversity, evenness and richness of each sample were determined using a Shannon diversity index, Simpson's evenness index and Chao1 index, respectively (Figure 2 and Supplementary Figure S4). Shannon diversity indices of samples with Benefiber and Psyllium husk reduced to 3.2 ± 0.5 and 2.4 ± 0.4, respectively, at 48 h, while samples with NutriKane showed no significant loss of diversity (3.8 ± 0.4) compared to the no added fiber control at 48 h (3.7 ± 0.2) and all samples at 0 h (4.0 ± 0.2). A similar trend was observed for the microbial evenness. Simpson's evenness indices for samples with Psyllium husk were significantly lower (0.71 ± 0.01) compared to the no added fiber control at 48 h (0.93 ± 0.04), while the microbial evenness of samples with NutriKane (0.92 ± 0.07) and Benefiber (0.89 ± 0.06) showed no significant loss of evenness. Microbial richness determined using a Chao1 index demonstrated no significant change in any of the treatments over time.

### Effects of fiber addition on microbiota composition

For all individuals the starting fecal microbiota communities (0 h) were dominated by the phyla *Firmicutes, Bacteroidetes, Actinobacteria, Proteobacteria*, and *Verrucomicrobia*. However, the relative abundances of these phyla differed substantially between biological samples. Similar individual-specific variations were observed at a family and genus level, and supplementation with each fiber product differentially altered the microbiota composition at 24 and 48 h in each of the six biological samples (Supplementary Figure S5).

The OTUs that contributed most to these product-specific changes in the microbial community composition were identified using SIMPER analyses, and changes in the relative abundance of this set of OTUs in each treatment and time point were analyzed for each biological sample (Supplementary Figure S6 and Supplementary Table S2). The OTUs in the genus *Bacteroides* showed a higher relative abundance in all samples supplemented with fiber, however, the specific OTUs varied between fiber products. In five out of six biological samples, the relative abundance of *Bacteroides* OTU589071 was significantly higher upon addition of Benefiber. While in at least four biological samples, the relative abundance of three OTUs (OTU364179, OTU535375 and OTU583117) in the genus *Bacteroides* were significantly higher with addition of Psyllium husk. In all biological samples, the relative abundance of two OTUs (OTU585914 and OTU180082) in the genus *Parabacteroides* were significantly higher upon addition of Benefiber. In three out of six biological samples, the relative abundance of *Coprococcus* (OTU362501) was significantly higher in samples with NutriKane compared to the no added fiber control, samples with Benefiber and Psyllium husk showed a reduction in the relative abundance of this OTU.

**Figure 3:**
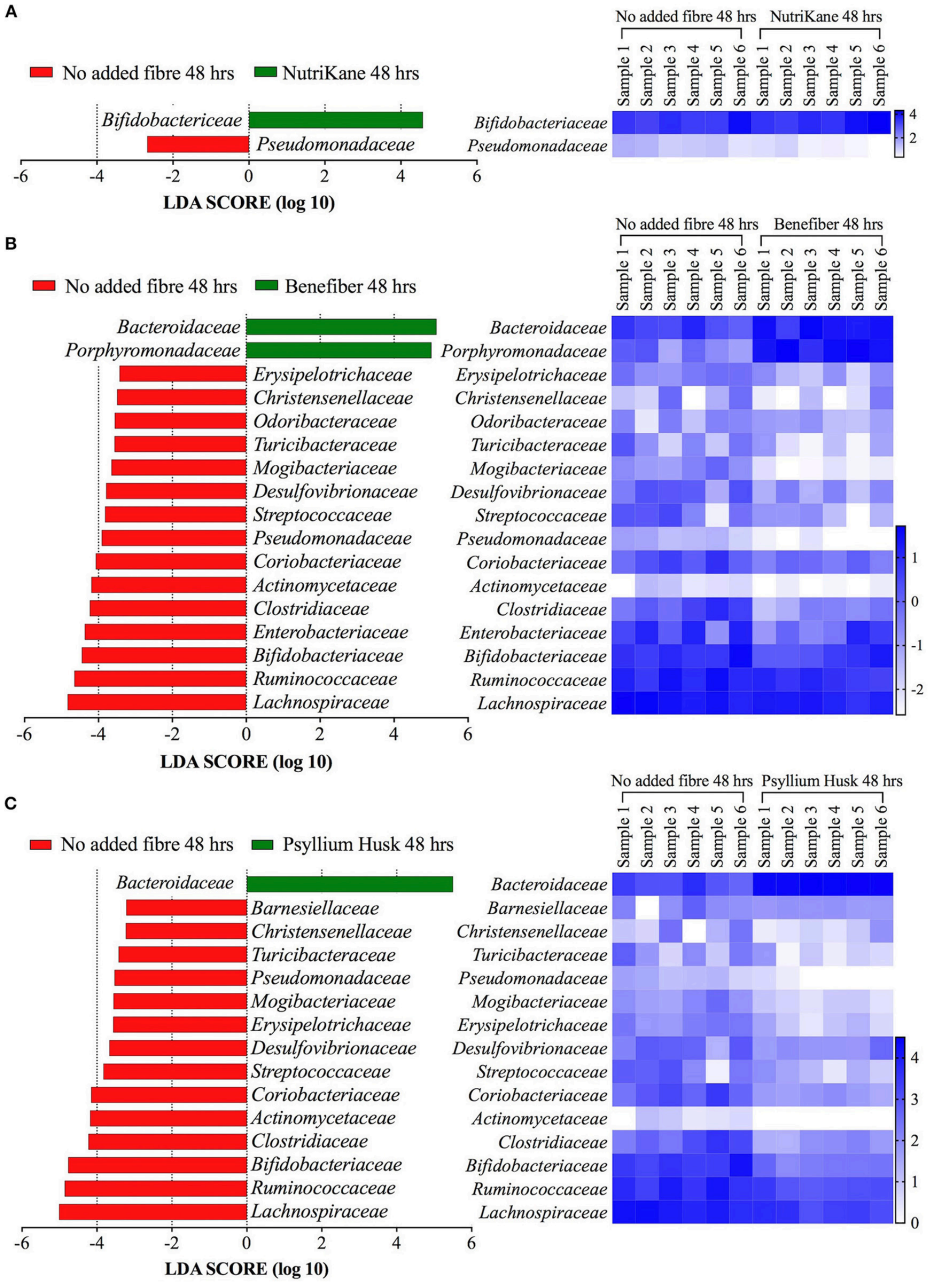
Key gut microbiota bacterial families that respond to fiber supplementation at 48 h. Data was obtained using LEfSe analyses between **(A)** NutriKane vs. no added fiber control, **(B)** Benefiber vs. no added fiber control and **(C)** Psyllium husk vs. no added fiber control. The left histogram shows the LDA scores computed for each bacterial family and the right heat map shows the relative abundance (Log_10_ transformation) of the families in each of the six biological samples. In the heat map, rows correspond to bacterial families and columns correspond to an individual (Sample 1–6). Blue and white denote the highest and lowest relative abundance, respectively, as shown in the key.

The complete set of bacterial families and OTUs showing significant differences in the relative abundance between each fiber addition and the no added fiber control was identified with LEfSe analyses. A family level LEfSe analysis showed 2, 17, and 15 differentially abundant families in NutriKane, Benefiber and Psyllium husk, respectively, compared to the no added fiber control (Figure 3). For NutriKane, the relative abundance of *Bifidobacteriaceae* and *Pseudomonadaceae* were shown to be significantly different. Benefiber and Psyllium husk supplementation resulted in an increase in the relative abundance of *Bacteroidaceae* compared to the no added fiber control. The relative abundance of the family *Porphyromonadaceae* significantly increased with addition of Benefiber. A decrease in the relative abundance of *Lachnospiraceae, Ruminococcaceae, Enterobacteriaceae* and *Bifidobacteriaceae* was observed upon supplementation with Benefiber and Psyllium husk.

The LEfSe analyses at the OTU level showed 72, 259, and 203 OTUs with significantly altered abundances in response to supplementation with NutriKane, Benefiber and Psyllium husk, respectively (Supplementary Table S3). While most of these trends were commonly observed across biological samples, the degree of changes varied between individuals (Supplementary Table S4). The relative abundance of many specific OTUs within the *Bacteroidaceae* were significantly higher in samples with each of the three fiber products compared to the no added fiber control (15, 35, and 33 OTUs in NutriKane, Benefiber and Psyllium husk, respectively, Supplementary Table S3). Among the fiber specific changes, Benefiber addition resulted in higher relative abundance of 5 and 15 OTUs in *Faecalibacterium prausnitzii* and *Parabacteroides* (of these, 8 OTUs were identified as *Parabacteroides distasonis*), respectively, a change not observed for the other products. NutriKane supplementation promoted high relative abundance of an OTU (OTU723) within the *Bifidobacteriaceae*, whereas supplementation with Benefiber and Psyllium husk decreased the relative abundance of 2 and 11 *Bifidobacteriaceae* family OTUs, respectively.

The relative abundance of OTUs in the *Enterobacteriaceae* decreased upon addition of all fiber products (2, 5, and 2 OTUs in NutriKane, Benefiber and Psyllium husk, respectively). The OTU646549 in the family *Pseudomonadaceae* also showed a lower abundance upon addition of each of the three fiber products. The relative abundance of many *Lachnospiraceae* OTUs decreased upon Benefiber and Psyllium husk supplementation (65 and 58 OTUs, respectively), whereas for NutriKane the relative abundance of 3 OTUs in this family decreased, while the abundance of 27 OTUs increased. Similarly, in the family *Ruminococcaceae* the relative abundance of 41 and 30 OTUs decreased in samples with Benefiber and Psyllium husk, while for NutriKane, the abundance of 11 OTUs in this family increased.

### Variation in the response of the biological samples to fiber supplementation

The addition of fiber products resulted in several common changes observed across most of the biological samples; including changes in the relative abundance of specific OTUs in the families *Bacteroidaceae, Porphyromonadaceae, Bifidobacteriaceae, Lachnospiraceae, Ruminococcaceae, Pseudomonadaceae*, and *Enterobacteriaceae* (Figure 3, Supplementary Figures S5, S6).

In addition to these, we also observed individual-specific changes in some bacterial groups (Supplementary Figure S5). Most notably, the relative abundance of the genus *Megamonas* increased dramatically only in biological sample 1 and 2 in the presence of Benefiber. In biological sample 2, *Butyricimonas* was highly abundant in the presence of all fiber products. The relative abundance of *Prevotella* showed dramatic changes only in biological samples 2 and 5 in the presence of Benefiber and Psyllium husk, respectively. The increase in the relative abundance of *Bifidobacterium* observed upon addition of NutriKane was substantially higher for biological sample 5 than for other biological samples. The abundance of *Bacteroidales* S24-7 was higher only in biological samples 1 and 6 following Psyllium husk and NutriKane treatments. The family *Comamonadaceae* was highly abundant upon addition of fiber products only in biological sample 4. In biological sample 5, the relative abundance of *Enterobacteriaceae* increased at 24 h upon addition of fiber products.

### Comparison of the fiber-adherent and liquid fraction microbiota

To investigate possible differences in the microbial communities adhered to the fiber relative to the liquid fraction, we examined the microbiota detached from insoluble material in the cultures at 48 h. While the community structure was observed to be similar between fiber and liquid fractions (Supplementary Figure S7), some differences in the composition were observed (Supplementary Figure S8). Analysis of the fiber adherent microbial community relative to the liquid fraction at the OTU level was performed using LEfSe analyses (Supplementary Table S5). The relative abundance of 13, 19, and 31 OTUs were higher in the fiber fraction of samples with NutriKane, Benefiber and Psyllium husk, respectively, most of these OTUs were in the families *Turicibacteraceae, Lachnospiraceae* and *Ruminococcaceae*. The relative abundance of 45, 44, and 24 OTUs were higher in the liquid fraction of NutriKane, Benefiber and Psyllium husk, respectively, most of these OTUs were in the families *Bacteroidaceae, Lachnospiraceae* and *Ruminococcaceae*. While most of these trends were commonly observed across biological samples, we again observed some individual-specific differences (Supplementary Table S6).

### Fiber additions increased the production of SCFAs

**Figure 4:**
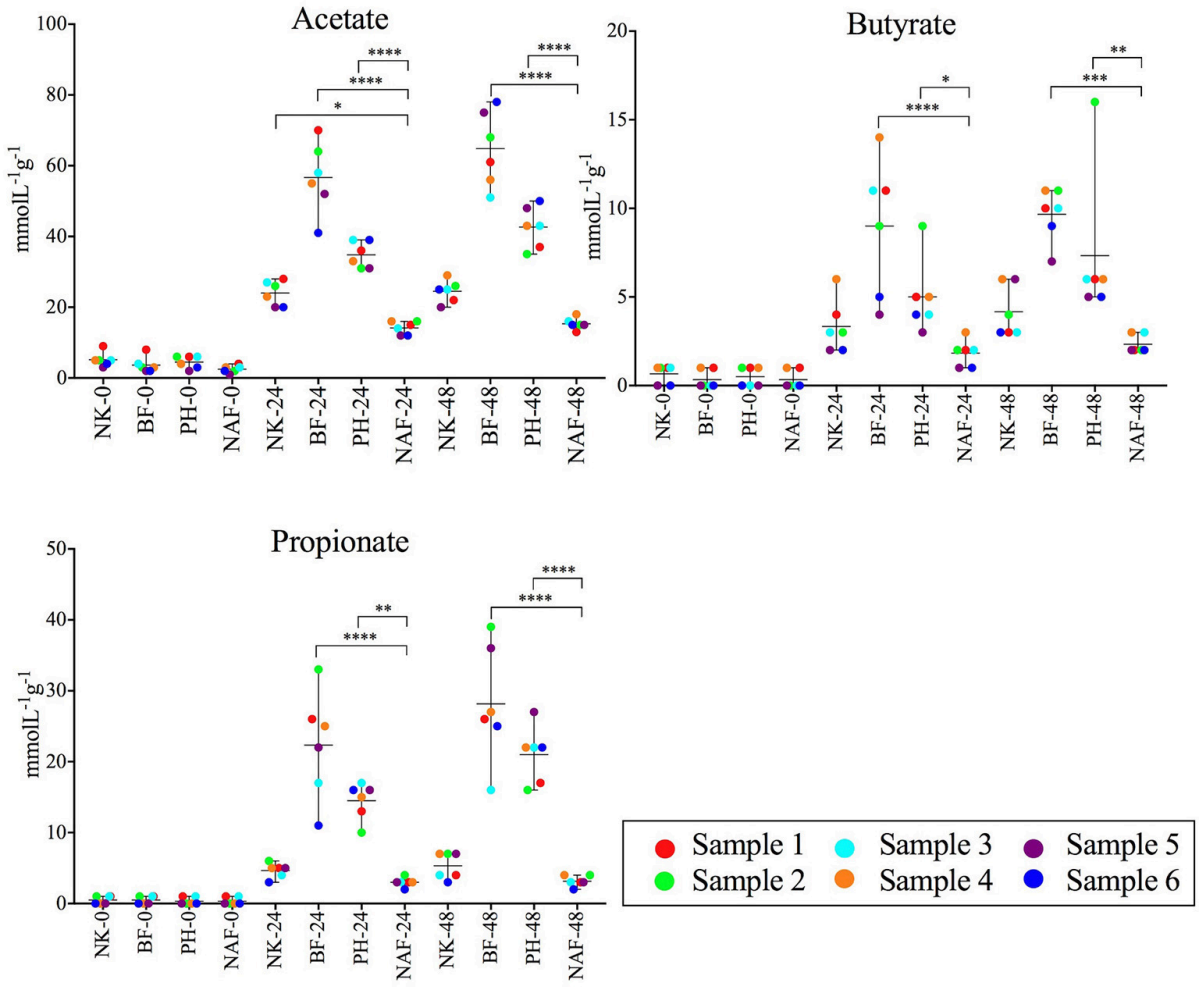
Average concentrations of acetate, butyrate and propionate in each sample at 0, 24, and 48 h. Concentrations of all the SCFAs increased upon the fiber additions (NK- NutriKane, BF- Benefiber, PH- Psyllium husk) at 24 and 48 h compared to the no added fiber control (NAF). The mean ± SD concentrations per treatment at 0, 24, and 48 h are shown. The mean concentration for each biological sample (sample 1–6) is indicated by color-coded dots as shown in the key. Significance was determined using Tukeys's multiple comparison tests (*P < 0.05, **P < 0.01, ***P < 0.001, ****P < 0.0001). The measured SCFA concentrations are provided in Supplementary Table S7.

To examine the impact of the fiber products on microbial production of SCFAs, the concentrations of acetate, propionate and butyrate were measured in the liquid fraction of the samples using a gas chromatograph with flame ionization detection (GC-FID, Figure 4). Addition of NutriKane, Benefiber and Psyllium husk resulted in significantly higher (*P* < 0.05) concentrations of all three SCFAs at 48 h, compared to the same treatments at 0 h. In comparison to the no added fiber control at 48 h, Benefiber and Psyllium husk supplementation showed significantly higher (*P* < 0.005) concentrations of all three SCFAs. The increase in concentration of all three SCFAs was the highest upon addition of Benefiber, followed by Psyllium husk and NutriKane. All fiber additions resulted in a significant increase in the concentrations of acetate, followed by propionate and butyrate. Samples with NutriKane had similar concentrations of propionate and butyrate at 48 h, while samples with Benefiber and Psyllium husk had two and three-fold higher concentrations of propionate relative to butyrate, respectively.

Changes in the relative abundance of the *Parabacteroides* correlated with the concentrations of all three SCFAs (Spearman's *r* > 0.33, *P* < 0.0001). Changes in the abundance of *Bacteroides* correlated with the concentration of propionate (Spearman's *r* = 0.43, *P* < 0.0001). While all biological samples showed similar trends with the specific fiber additions, we observed individual-dependent differences in the concentrations of each SCFA (Supplementary Table S7).

### Comparison of the polyphenol content and antioxidant potential of fiber products

**Figure 5:**
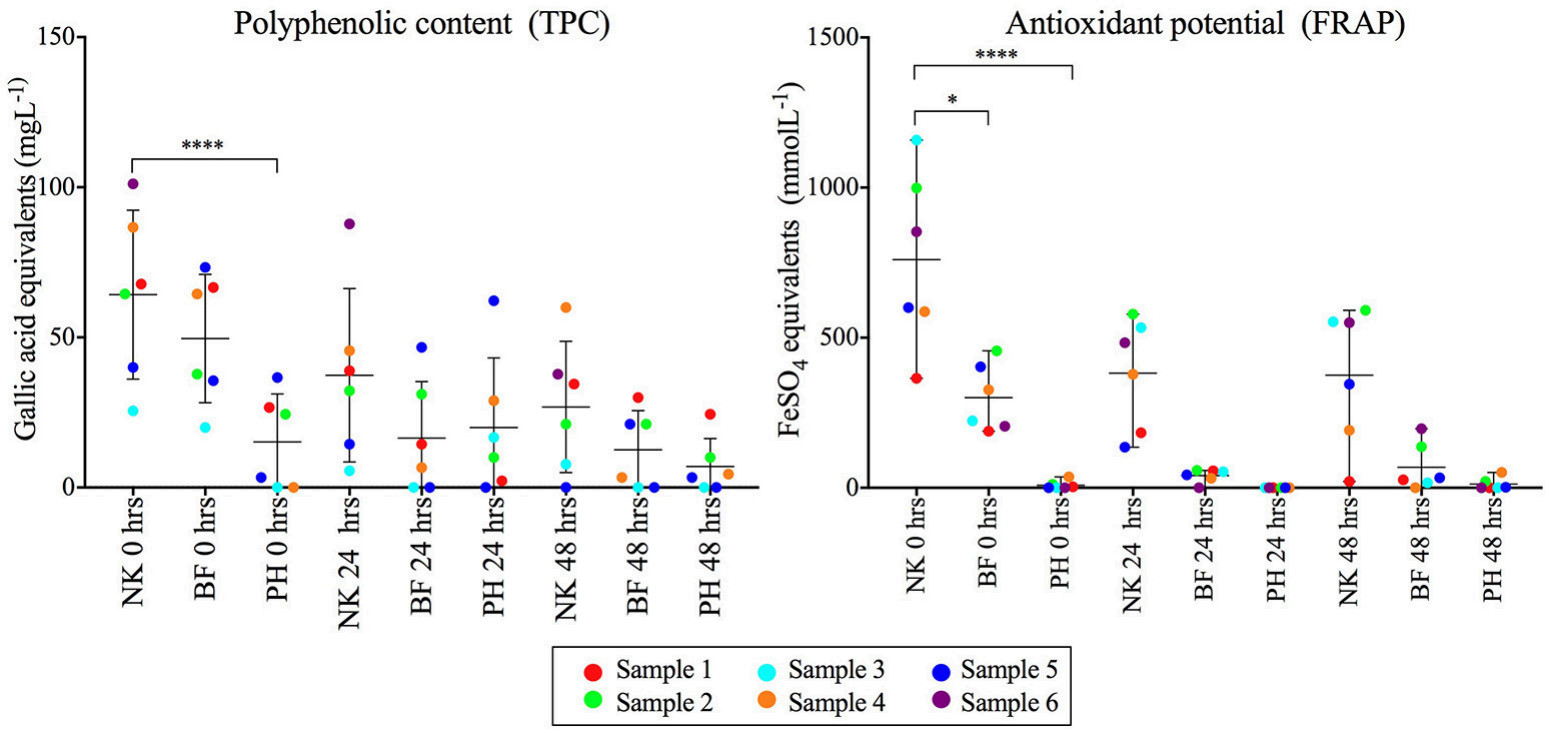
Comparison of the polyphenol content (mgL^−1^) and antioxidant potential (mmolL^−1^) of the fiber products (NK- NutriKane, BF- Benefiber, PH- Psyllium husk) in each sample at 0, 24 and 48 h. Measurements for each fiber addition in each biological sample have been normalized against the no added fiber control at equivalent time points. Data is shown as mean ± SD for each treatment at 0, 24, and 48 h. Biological samples are indicted by color-coded dots as shown in the key. Significance was determined using Kruskal-Wallis test with Dunn's multiple comparisons (*P < 0.05, ****P < 0.0001). The measured concentrations are provided in Supplementary Table S8.

The polyphenolic content and antioxidant potential of each fiber product at time 0, 24, and 48 h were determined using total polyphenolic content (TPC) and Ferric reducing antioxidant power (FRAP) techniques respectively (Figure 5, Supplementary Table S8). NutriKane showed significantly higher (*P* < 0.0001) antioxidant potential and polyphenolic content compared to Psyllium husk at 0 h. The antioxidant potential of NutriKane was significantly higher (*P* < 0.05) compared with Benefiber at 0 h. In all fiber-supplemented samples polyphenolic content decreased across the full incubation while antioxidant potential decreased over the first 24 h, but no further decrease was observed at 48 h.

## Discussion

This research examined the effect of three commercially available fiber products on the human gut microbiota from healthy individuals *in vitro*. Our findings demonstrated fiber product-induced strong shifts in gut microbiota community structure and composition at 24 h, which were further pronounced at 48 h. These changes in the relative abundance of microbial families and OTUs following fiber additions are largely consistent with selection for the abundance of bacteria capable of polysaccharide digestion. Amongst the most highly stimulated families were groups known to produce high numbers of Carbohydrate-active enzymes (CAZymes) that contribute to the digestion of polysaccharides by the gut microbiota. This includes specific members of the phyla *Bacteroidetes* such as *Bacteroidaceae* and *Porphyromonadaceae*, reported to encode the highest number of CAZymes, or members of the *Firmicutes* (*Lachnospiraceae* and *Ruminococcaceae*) and *Actinobacteria* (*Bifidobacteriaceae*), which are also CAZyme-producing (El Kaoutari et al., 2013).

### Tested fiber products have distinct chemical compositions

NutriKane is primarily derived from dried whole sugarcane stem and pectin, while Benefiber and Macro Psyllium husk is produced using wheat dextrin and psyllium husk, respectively. Composition analysis of the three fiber products demonstrated distinct chemical profiles in dietary fiber, carbohydrates, protein and fat content for each product. The amount of lignin and xylose was highest in NutriKane, while Benefiber had the highest amounts of mannose and glucose, and Psyllium husk contained the highest amount of arabinose. Previous studies have reported sugarcane to be rich in cellulose, hemicellulose and lignin (Ouensanga, 1989; Hoang et al., 2016), and contain a range of β-1, 4, and α-1, 4 linkages between glucose, xyloglucans, xylans, glucomannan, arabinoxylan, glucuronoxylan and D-galacturonic acid (Sun et al., 2003, 2004; Scheller et al., 2010; Hemsworth et al., 2016). Wheat dextrin contains typical starch glucosidic bonds (α-1, 4, and α-1, 6) and bonds atypical of starch (α-1, 2, and α-1, 3) between D-glucose subunits (Noack et al., 2013; McRorie, 2015). Psyllium husk typically contains viscous fiber such as certain hemicelluloses and arabinoxylans, and has been reported to consist of densely substituted main chains of β-1, 4 linked D-xylopyranosyl residues (Fischer et al., 2004). According to our results, NutriKane had the highest amount of total dietary fiber, followed by Psyllium husk and Benefiber, this observation is different from the information provided on the packaging. However, the testing applied here followed a standard protocol, which is less effective for lower molecular weight products and therefore may have potentially underestimated the soluble dietary fiber fraction for Benefiber in particular. As there is currently no universally applied protocol required for determining package labeling nutritional information for Australian dietary fiber products, the protocols used to generate the packaging information are not known and may not have been consistent across products, highlighting the need for standardized protocols.

### Each fiber product resulted in distinct alterations to the microbiota composition

Fiber-specific microbial community changes were observed, which are potentially linked to the chemical composition of each tested product. The abundance of OTUs in *Bacteroidaceae* (genus *Bacteroides*) and *Porphyromonadaceae* (genus *Parabacteroides*) were significantly higher upon addition of Benefiber and Psyllium husk, whilst OTUs in *Lachnospiraceae* and *Ruminococcaceae* were highly abundant in the presence of NutriKane. The members in the genus *Parabacteroides* (*P. distasonis* in particular) have been shown to digest chemically modified starch, while members in *Bacteroides, Ruminococcaceae* and *Lachnospiraceae* digest starch as well as more complex polysaccharides such as cellulose, hemicellulose and pectin (Martínez et al., 2010; Cockburn and Koropatkin, 2016). An increase in the abundance of *Bifidobacterium, Lactobacilli, Roseburia* and *Clostridium* cluster XIVa upon addition of wheat dextrin has been previously reported (Hobden et al., 2013; Noack et al., 2013; Carlson et al., 2015), whilst an increase in the relative abundance of *Bacteroides* and *Parabacteroides* has not been previously reported. Previous studies have also investigated the effect of specific purified dietary fibers on the gut microbiota and have shown that different types of resistant starch (Martínez et al., 2010), pectin (Licht et al., 2010), hemicellulose (Sanchez et al., 2009), cellulose (Chassard et al., 2010) and inulin (Van de Wiele et al., 2007) have different effects on the gut microbiota, likely due to variations in the chemical composition of different dietary fibers (Hamaker and Tuncil, 2014). In addition to differences in the dietary fiber and carbohydrate profiles between NutriKane, Benefiber and Psyllium husk, differences in the protein and fat content could also have contributed to observed fiber product-specific gut microbiota changes. The relative abundance of the OTUs in the genera *Bacteroides* was significantly higher in samples with Psyllium husk. This product had the highest amount of protein, consistent with the ability of the members of the genus *Bacteroides* to metabolize dietary protein (Portune et al., 2016).

Significant increases in the relative abundance of *Bifidobacteriaceae* (genus *Bifidobacterium*) were observed solely for NutriKane. NutriKane contained higher levels of xylose, and *Bifidobacteriaceae* has been shown to cross-feed on xylan (Cockburn and Koropatkin, 2016). Increased relative abundance of *Bifidobacterium* has been linked to potential prebiotic effects and has been shown to increase in IBD patients upon remission (Morgan et al., 2012; Papa et al., 2012; Delzenne et al., 2013; Hamaker and Tuncil, 2014). *Faecalibacterium prausnitzii* showed higher relative abundance following supplementation with Benefiber. This is potentially linked to the ability of this group to digest smaller carbohydrates such as glucose (Cockburn and Koropatkin, 2016), which are highly available in this product. Increases in this species have been observed to have potential anti-inflammatory effects on patients with Crohn's disease (Sokol et al., 2008).

The OTUs in the families *Enterobacteriaceae* and *Pseudomonadaceae* showed the highest relative abundance in samples with no added fiber. Most of the members of these families belong to the normal microbiota, while some are associated with inflammation (Morgan et al., 2012; Shin et al., 2015). Observed reductions in the relative abundance of *Enterobacteriaceae* and *Pseudomonadaceae* upon supplementation with these fiber products might indicate the potential of the products to improve or maintain host health.

Analysis of the diversity and evenness of each community after fiber addition indicated varied capacities of the fiber products to maintain gut microbiota diversity and evenness *in vitro*. Of the tested fibers, only NutriKane treatment resulted in maintenance of the microbial diversity, while supplementation with Benefiber and Psyllium husk resulted in significant reductions. The Simpson evenness indices reflected the same observation whereby the microbial evenness was significantly lower in samples with Psyllium husk. These are likely explained by the dramatic increases in fiber-digesting groups such as *Parabacteroides*, which constituted 30.5% of the total bacteria at 48 h in Benefiber, and the *Bacteroides*, which constituted 68.9 and 24.0% of the total bacteria at 48 h in the samples with Psyllium husk and Benefiber, respectively. Whilst reduced gut microbiota diversity has been shown in individuals with obesity, T2D and IBD (Ott, 2004; Le Chatelier et al., 2013), we believe such dramatic increases in specific groups would likely be ameliorated by host factors, the presence of other fiber and food components and phage controls *in vivo*. Hence the reduction in diversity and evenness observed in the *in vitro* system is unlikely to be observed *in vivo*.

### Microbiota composition differed between fiber-adherent and liquid fractions

The relative abundance of bacterial groups attached to the insoluble fiber fraction, and therefore potential primary degraders, varied between each tested fiber product. The relative abundance of members of the phyla *Firmicutes* (*Lachnospiraceae* and *Ruminococcaceae*), and *Verrucomicrobia* (*Turicibacteraceae*) were higher in the fiber fraction compared to the liquid fraction. Bacterial groups in the phyla *Firmicutes, Actinobacteria* and *Verrucomicrobia* are known to be more nutritionally specialized and initiate the degradation of complex carbohydrates undigested by human (Flint et al., 2012) and, as primary degraders, may be preferentially found attached directly to the complex polysaccharides in the tested products. Previous studies have also demonstrated that the microbial communities attached closely to the insoluble material in human fecal samples are different to the liquid fraction communities, potentially due to the ability of the communities adhered to the insoluble material to act as primary degraders (Walker et al., 2008; White et al., 2014).

### Fiber additions stimulated production of acetate, propionate, and butyrate

The concentration of measured SCFAs was significantly higher in all fiber-supplemented samples. Acetate, propionate and butyrate are all major bacterial fermentation products, each of which is likely to contribute to host health (Koh et al., 2016). Butyrate is generally used as an energy source in the colonic epithelial cells, while acetate and propionate have been shown to reach the liver and other peripheral organs and contribute in regulating gluconeogenesis and lipogenesis (Tremaroli and Backhed, 2012). The degree of stimulation of SCFA production was fiber product dependent and was more pronounced following addition of Benefiber and Psyllium husk compared to NutriKane. High levels of SCFA production in the presence of Benefiber and Psyllium husk correlated with the higher relative abundance of *Parabacteroides* and *Bacteroides* following addition of these fiber products. This is potentially linked to the ability of the members of these bacterial genera to digest highly available dietary fiber in the fiber products, and produce SCFAs (Kelder et al., 2014).

While production of all three SCFAs was higher with fiber additions, the pH levels significantly reduced only in samples with Benefiber. The significantly higher SCFA production in Benefiber supplemented samples may have surpassed the buffering capacity of the medium, while buffering was maintained in other samples where SCFA production was lower. Metabolic activities of SCFA-producing bacteria have been previously reported to reduce the pH of the large intestine, and lower intestine pH levels has also been linked to inhibit the growth of pathogenic *Escherichia coli* (Duncan et al., 2009).

### Polyphenol and antioxidant availability differed between fiber products

We observed significant differences in the availability of polyphenols and antioxidants in the tested fiber products. Such differences may contribute to the product-specific changes observed in the gut microbiota. Dietary polyphenols have the potential to be used by the gut microbiota, and therefore, alter the microbial composition both *in vitro* (Tzounis et al., 2008; Condezo-Hoyos et al., 2014) and *in vivo* (Tzounis et al., 2011). Of the tested fiber products, NutriKane showed the highest availability of polyphenols and antioxidant potential. This could potentially have contributed to the higher relative abundance of the family *Bifidobacteriaceae* in samples with this particular fiber, as previous literature has shown an increase in the abundance this family upon addition of various polyphenol extracts and polyphenol rich foods both *in vitro* and *in vivo* (Hervert-Hernández and Goñi, 2011; Tzounis et al., 2011). The observed reduction of polyphenols and antioxidant potential of the fiber products over the time of incubation is likely due to metabolism of these compounds by the gut microbiota (Dueñas et al., 2015; Ozdal et al., 2016).

### Fiber supplementation-induced biological sample-specific microbial community shifts

In addition to common microbiota changes observed across biological samples, several sample-specific alterations were also observed, especially with bacterial groups such as *Megamonas, Butyricimonas, Bifidobacterium, Bacteroidales* S24-7, *Comamonadaceae* and *Prevotella*, which showed comparatively high relative abundances in some biological samples at 48 h while other biological samples did not show substantial differences. Of these, *Megamonas, Bacteroidales* S24-7, *Comamonadaceae* and *Prevotella* were not present or present at a very low relative abundance in all other biological samples except the specific samples that showed an increase in the relative abundance of these groups at 48 h. This may indicate that individual-specific differences are likely linked to the differences in the initial gut microbiota composition between the samples. Analysis of a larger number of biological samples, ideally with greater information on normal diet and host health, might be beneficial in determining possible reasons for this variability.

## Conclusions

As dietary supplementation grows in popularity it is important to examine how commercial fiber products impact the human gut microbial communities and host health, and the degree to which this varies between products. We tested the *in vitro* impact of three different fiber supplements using fecal microbial communities sourced from six healthy individuals. For each specific fiber a broad pattern was observed across the biological samples with respect to changes in the microbial community composition and concentrations of SCFAs. Underlying this, in specific individual samples there were variations in the precise nature of the fiber-induced microbiota community shifts likely linked to differences in the starting microbial communities. This suggests that each of the tested fiber products may alter the gut microbiota in a generally similar, and rather predictable manner, despite variability in the starting composition of the individual gut microbiota.

The three different fiber products tested in this study all showed clear and distinct impacts on the structure and composition of the microbiota derived from healthy individuals. Differences in the impact on the microbiota could be linked to the composition of the dietary fiber and its associated micronutrients, for example the antioxidant and polyphenol content in each fiber product. The observed differences in microbial community composition upon fiber supplementation may also explain the observed fiber-specific differences in acetate, propionate and butyrate production.

Utilization of an *in vitro* gut mimicking model system in the present study facilitated frequent sampling without host interference and provided proof of concept information on how dietary fiber supplementation may influence the microbiota composition and function. To follow up, *in vivo* experiments could be conducted to gain further insight into the long-term effect of fiber products on the gut microbiota and how long the benefits last after consumption, while also taking differences in health, normal diet and colonic transit time between individuals into account (Verspreet et al., 2016).

## Availability of data and material

The 16S rRNA gene sequence data generated and analyzed for this study can be found in the GenBank Sequence Read Archive (SRA) database under accession number SRP090829.

## Author contributions

HG, ST, IP, and CR designed the study. HG conducted *in vitro* digestion, culturing, DNA extraction, bioinformatics and all statistical analyses. RC quantified SCFA concentrations and determined the chemical composition of fiber products. DB-N performed TPC and FRAP quantifications. HG, ST, IP, LK, MB, MM, and NP interpreted the results. HG drafted the manuscript with contributions of DB-N, RC, ST, and IP. All authors read and approved the final manuscript.

### Conflict of interest statement

MB is an employee of Gratuk Technologies Pty Ltd, producer of NutriKane^TM^. The remaining authors declare that the research was conducted in the absence of any commercial or financial relationships that could be construed as a potential conflict of interest.
