# Supplementary material for: Fiber Supplements Derived From Sugarcane Stem, Wheat Dextrin and Psyllium Husk Have Different In Vitro Effects on the Human Gut Microbiota
Source: Front Microbiol. 2018 Jul 20;9:1618. doi: 10.3389/fmicb.2018.01618 (PMC6060387; doi:10.3389/fmicb.2018.01618)
Supplement: Supplementary file 1 [file Image_1.PDF]

Supplementary material

Fibre Supplements Derived From Sugarcane Stem, Wheat Dextrin And Psyllium Husk Have Different *In Vitro* Effects On The Human Gut Microbiota

Hasinika K.A.H. Gamage[1,2], Sasha G. Tetu[1*], Raymond W.W. Chong[1,2], Daniel Bucio-Noble[1,2], Carly P. Rosewarne[3,4], Liisa Kautto[1,2], Malcolm S. Ball[5], Mark P. Molloy[1,2], Nicolle H. Packer[1,2], Ian T. Paulsen[1,2*]

[*]Corresponding authors: Ian T. Paulsen, ian.paulsen@mq.edu.au and Sasha G. Tetu, sasha.tetu@mq.edu.au.

Supplementary Figures and table legends

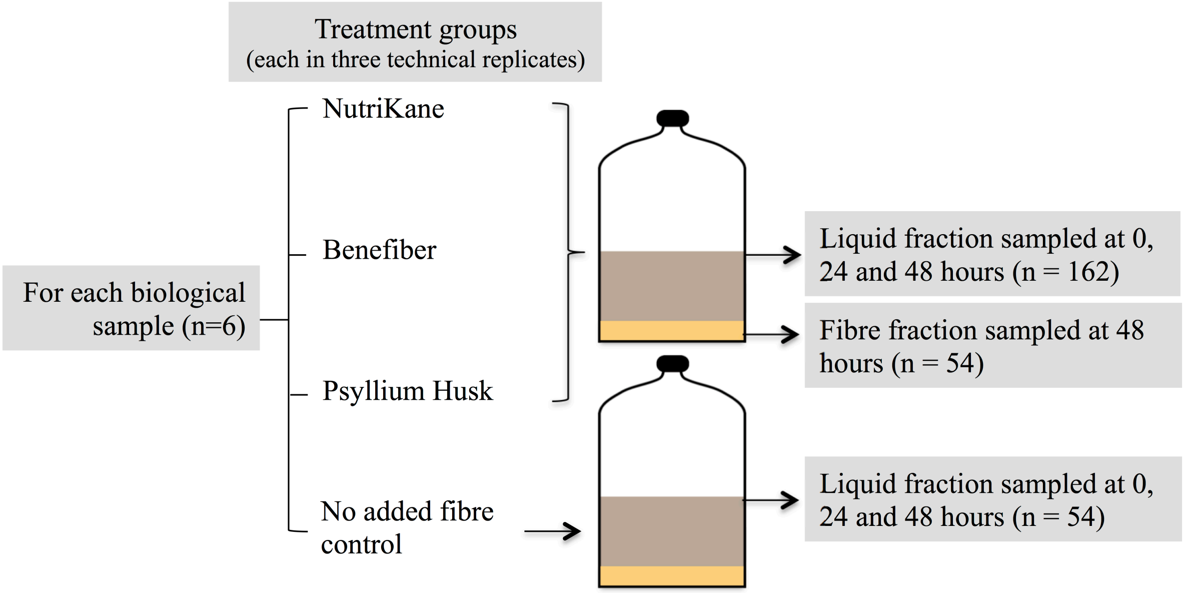

Supplementary Figure S1 Experimental design. Fecal material obtained from six healthy adults as independent biological samples (n=6) were inoculated separately into the basal medium. For each biological sample, four treatments were applied, this included three fibre products (NutriKane, Benefiber and Psyllium husk) and one ‘no added fibre’ control. Top liquid fraction of each culture was sampled at 0, 24 and 48 hours of incubation. At 48 hours the insoluble fibre fraction of cultures with the three fibre products were sampled separately. This resulted in a total of 216 samples from the liquid fraction and 54 samples from the fibre fraction.

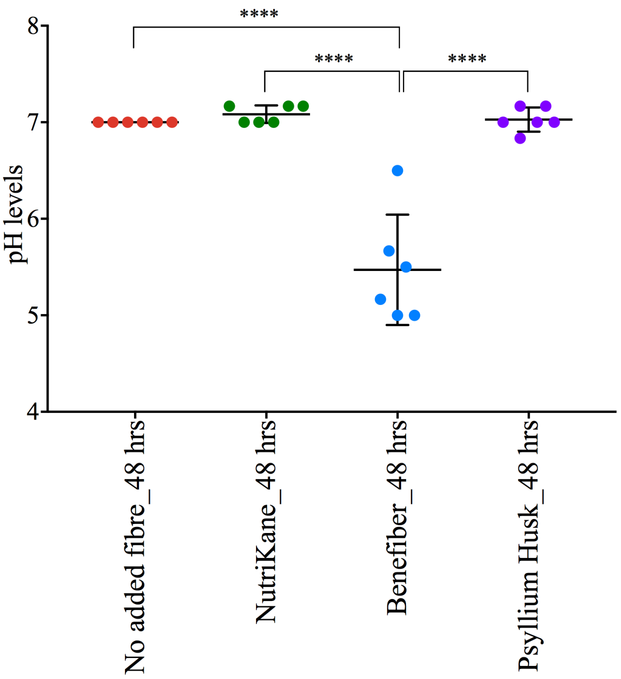

Supplementary Figure S2 Measurements of pH for all cultures at 48 hours. Mean pH values for each of the three technical replicates in each of the six biological samples are indicated (dots). Bars represent the mean pH levels with ± SD for each treatment. Significance was determined using Kruskal-Wallis test with Dunn's multiple compassions (**** $P < 0.0001$).

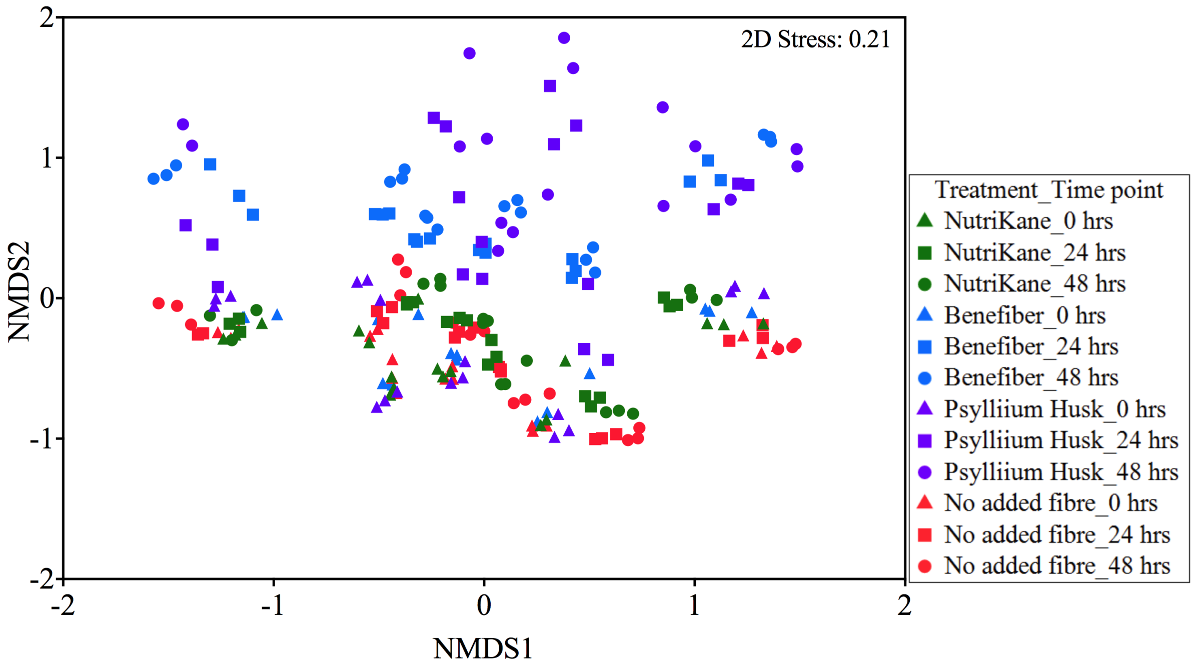

Supplementary Figure S3 Bray-Curtis similarity based nMDS plot indicating the ordination of the gut microbiota at 0, 24 and 48 hours for all six individuals. All biological samples showed similar fibre-dependent shifts in the microbial community structure. Samples at 0 hours were grouped together independent of fibre addition. Fibre-dependent shifts were observed at 24 and 48 hours.

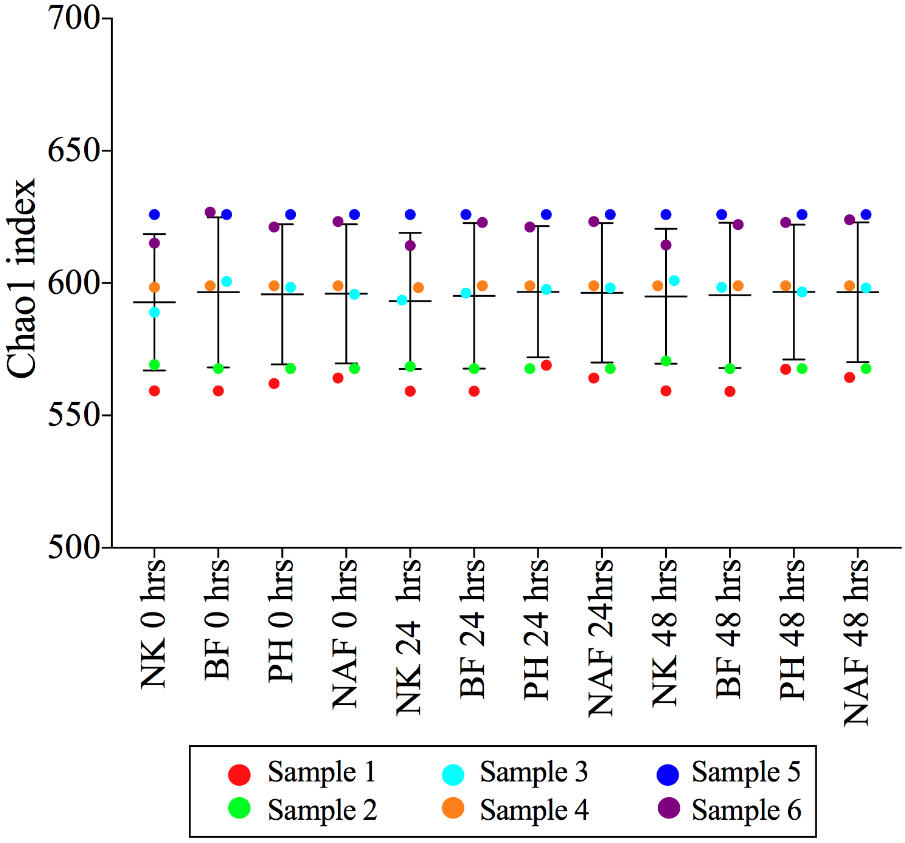

Supplementary Figure S4 The Chao1 indices of microbial communities from each treatment at 0, 24 and 48 hours. Data is shown as mean ± SD for samples with NutriKane (NK), Benefiber (BF), Psyllium husk (PH) and no added fibre control (NAF) at 0, 24 and 48 hours. Biological samples (sample 1-6) are indicted by colour-coded dots as shown in the key.

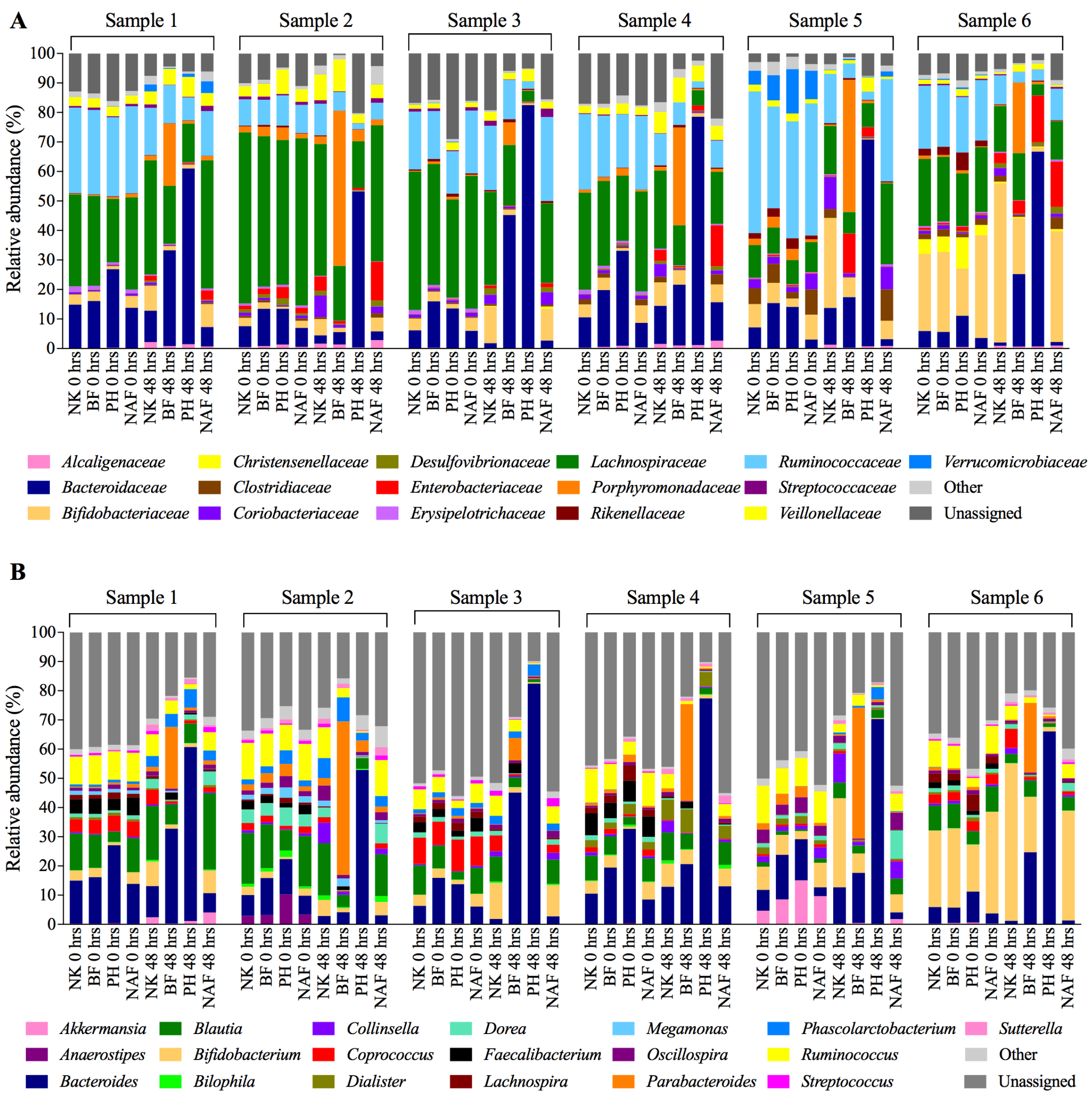

Supplementary Figure S5 (A) Family and **(B)** genus level taxonomic composition of the microbial communities across treatments at 0 and 48 hours for each biological sample (Sample 1-6). The relative abundance of 16S rRNA gene amplicons in the families and genera were determined using QIIME and graphed using GraphPad Prism (Version 7). Bacterial identifications that were not assigned to a family or genus are categorised as "Unassigned". Bacterial groups with a relative abundance < 2% in all the treatments at all time points are indicated as "Other". Column labels are abbreviated as, NK- NutriKane, BF- Benefiber, PH- Psyllium husk and NAF- No added fibre control at 0, 24 and 48 hours.

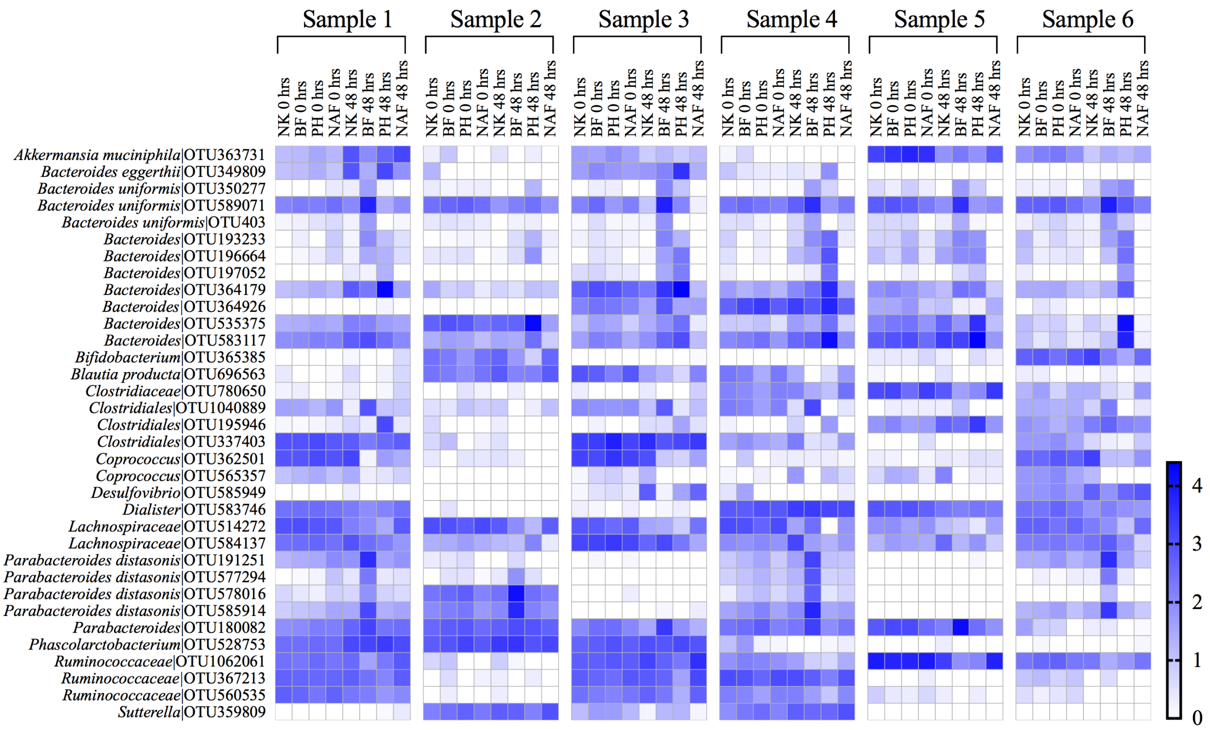

Supplementary Figure S6 Fibre-dependent changes in the relative abundance of differentially abundant OTUs in each treatment at 0 and 48 hours. This subset of OTUs were shown to have different relative abundances in each fibre treatment at 48 hours compared to the no added fibre control at equivalent time points, based on SIMPER analysis. Plotted data is Log_{10} transformed relative abundance of selected OTUs (rows) for each treatment condition per biological sample (Sample 1-6). The highest possible taxonomic identification is given before each OTU number. Blue and white denote highest and lowest relative abundance, respectively. The intensity of colours represents the level of the abundance as shown in the key. Columns are each fibre product treatment at 0 and 48 hours abbreviated as, NK- NutriKane, BF- Benefiber, PH- Psyllium husk and NAF- No added fibre control. The relative abundances and significance of these OTUs are provided in Table S2

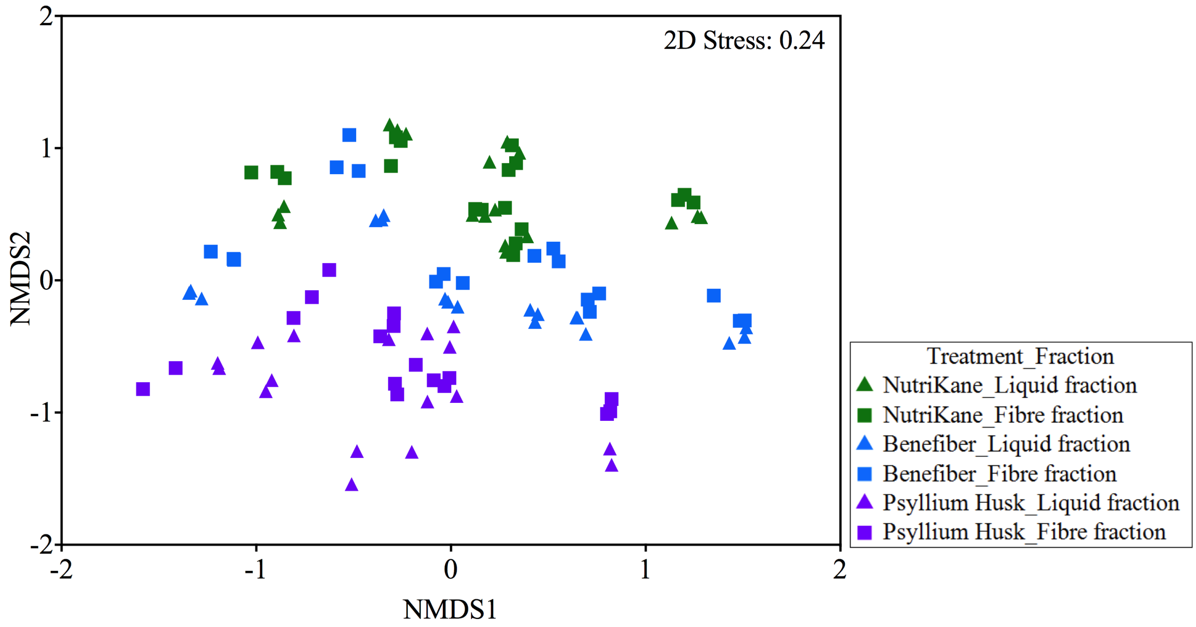

Supplementary Figure S7 Bray-Curtis similarity based nMDS plot indicating the ordination of the fibre-adherent and liquid gut microbiota fractions. The community of bacteria adhered to the fibre material was compared to that of the liquid fraction. The community structure between the fibre and liquid fraction were similar in all fibre products.

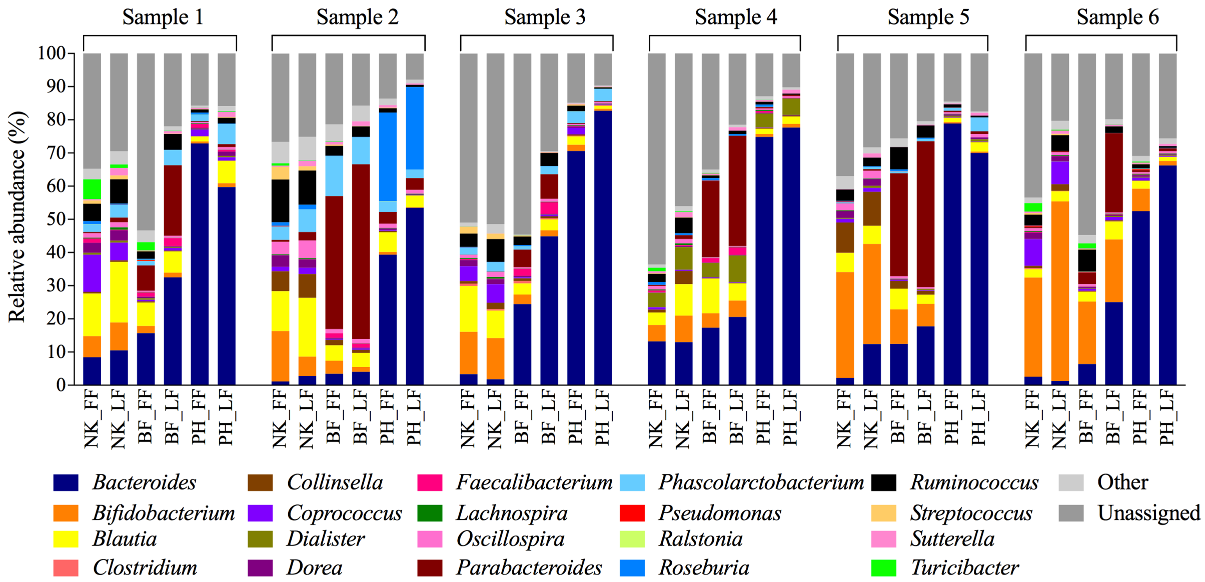

Supplementary Figure S8 Differences in the bacterial relative abundance between the fibre fraction (FF) and liquid fraction (LF) microbiota at the genus level. Data is shown for each biological sample (sample 1-6) with each fibre product (NK- NutriKane, BF- Benefiber, PH- Psyllium husk) at 48 hours. Bacterial identifications that were not assigned to a genus are categorised as “Unassigned”. Bacterial groups with a relative abundance < 2% in all the treatments at all the time points are indicated as “Other”.

Supplementary Table legends

Supplementary Table S1 Metadata of the six biological samples (sample 1-6). None of the volunteers had consumed antibiotics in at least three weeks prior to sample submission. All individuals consumed a non-specific omnivorous diet and had no existing medical conditions.

Supplementary Table S2 The relative abundance of specific OTUs that were found to contribute to fibre-specific microbiota alterations, based on SIMPER analysis. Mean ± SD for samples with NutriKane (NK), Benefiber (BF), Psyllium husk (PH) and no added fibre control (NAF) at 0 and 48 hours for each biological sample (sample 1-6) is shown. Significance was determined using Tukey's multiple comparisons tests with * $P < 0.05$, ** $P < 0.01$, *** $P < 0.001$ and **** $P < 0.0001$ comparing fibre addition to the no added fibre control.

Supplementary Table S3 The OTUs that were significantly differentially abundant between each product and the no added fibre control at 48 hours. Data were obtained using LEfSe analysis between **(A)** NutriKane vs no added fibre control, **(B)** Benefiber vs no added fibre control and **(C)** Psyllium husk vs no added fibre control. The key OTUs with the taxonomic identifications and LDA scores are provided.

Supplementary Table S4 The abundance of the OTUs in cultures with each of the fibre additions (NutriKane (NK), Benefiber (BF) and Psyllium husk (PH)) and the no added fibre control (NAF) at 0, 24 and 48 hours (n=212). Data is shown for each biological sample (Sample 1-6).

Supplementary Table S5 The OTUs that were significantly differentially abundant between the fibre adherent and liquid fraction microbiota in cultures with each fibre product at 48 hours. Data were obtained using LEfSe analysis between the fibre adherent and liquid fraction microbiota of **(A)** NutriKane **(B)** Benefiber and **(C)** Psyllium husk. The key OTUs with the taxonomic identifications and LDA scores are provided.

Supplementary Table S6 The abundance of the OTUs in the fibre (FF) and liquid fraction (LF) microbiota of cultures with NutriKane (NK), Benefiber (BF) and Psyllium husk (PH) at 48 hours. Data is shown for each biological sample (Sample 1-6).

Supplementary Table S7 SCFA concentrations of each sample. Values are the average concentrations of the technical triplicates performed for each sample obtained across all the treatments, biological samples (Sample 1-6) and technical replicates. All values are expressed in $mmolL^{-1}g^{-1}$ with SD. ND = Not detected.

Supplementary Table S8 Antioxidant potential ($mmolL^{-1}$) and Polyphenolic content (mgL^{-1}) measurements for each biological sample (Sample 1-6). Measurements have been normalised against the no added fibre control. Mean values ± SD for technical replicates of NutriKane (NK), Benefiber (BF) and Psyllium husk (PH) at 0, 24 and 48 hours are provided.
